# The Chemical Profile of *Senna velutina* Leaves and Their Antioxidant and Cytotoxic Effects

**DOI:** 10.1155/2016/8405957

**Published:** 2016-10-10

**Authors:** Jaqueline Ferreira Campos, David Tsuyoshi Hiramatsu de Castro, Marcio José Damião, Heron F. Vieira Torquato, Edgar J. Paredes-Gamero, Carlos Alexandre Carollo, Leticia M. Estevinho, Kely de Picoli Souza, Edson Lucas dos Santos

**Affiliations:** ^1^School of Environmental and Biological Science, Federal University of Grande Dourados, Rodovia Dourados Itahum, Km 12, 79.804-970 Dourados, MS, Brazil; ^2^Department of Biochemistry, Federal University of São Paulo, Rua Pedro de Toledo 669, 04039-032 São Paulo, SP, Brazil; ^3^Interdisciplinary Center of Biochemistry Investigation, University of Mogi das Cruzes, Av. Dr. Cândido Xavier de Almeida Souza, No. 200, Mogi das Cruzes, SP, Brazil; ^4^Center of Biological and Health Sciences, Federal University of Mato Grosso do Sul, Campo Grande, MS, Brazil; ^5^Department of Biology and Biotechnology, Agricultural College of Bragança, Polytechnic Institute of Bragança, Campus Santa Apolónia, 5301-855 Bragança, Portugal

## Abstract

Natural products can be a source of biomolecules with antioxidant activity which are able to prevent oxidative stress-induced diseases and show antitumor activity, making them important sources of new anticancer drug prototypes. In this context, this study aimed to analyze the chemical composition of an ethanol extract of* Senna velutina* leaves and to assess its antioxidant and cytotoxic activities in leukemic cells. The antioxidant properties were evaluated using a DPPH free radical scavenging assay and by examining the extract's inhibition of AAPH-induced lipid peroxidation in human erythrocytes. Its cytotoxicity and possible mechanisms of action were assessed in Jurkat and K562 leukemic cell lines. The ethanol extract contained flavonoids, such as epigallocatechin, epicatechin, kaempferol heteroside, rutin, and dimeric and trimeric proanthocyanidin derivatives. The extract exhibited antioxidant activity by scavenging free radicals and antihemolytic action, and it decreased malondialdehyde content in human erythrocytes. Furthermore, the extract also induced leukemic cell death by activating intracellular calcium and caspase-3, decreasing mitochondrial membrane potential, and arresting the cell cycle in S and G2 phases. Hence,* S. velutina* leaf extract contains antioxidant and antileukemic biomolecules with potential applications in diseases associated with oxidative stress and in the inhibition of tumor cell proliferation.

## 1. Introduction

Several diseases, including cancer, diabetes, atherosclerosis, inflammatory diseases, and premature aging, are related to oxidative stress [1]. Oxidative stress stems from excess of free radicals in the body and low antioxidant activity, resulting in damage to essential biomolecules such as nucleic acids, proteins, and lipids [2].

Cancer is an oxidative stress-related disease that causes high rates of morbidity and mortality in the global population [3]. Leukemias are cancers that affect the cells of the hematopoietic system; depending on their cellular origin and maturity stage, leukemias can be classified as either myeloid or lymphoid and as acute or chronic [4]. Surgery, radiotherapy, and chemotherapy [5] are among the main types of treatment for these cancers.

Biomolecules with anticancer activity at low therapeutic doses and with reduced side effects have been increasingly sought in recent decades [6]. Between 1940 and 2014, 49% of the 174 anticancer drugs that were made available on the market were either natural products or their derivatives [7]. Thus, there is a trend among the general population and the medical community to regard medicinal plants as alternative sources of antitumor drugs, provided that the therapeutic properties of such plants have been scientifically researched and proven [8]. The discoveries of paclitaxel [9], an anticancer drug, and of homoharringtonine [10], which is used in the treatment of acute and chronic myeloid leukemia, are examples of successful cases in the development of medicinal plant-derived drugs.

The search for new molecules with therapeutic properties, including antioxidant and anticarcinogenic activities, is facilitated by the vast biodiversity and bioprospecting potential in Brazil.* Senna* genus has been used in Brazilian folk medicine for its antioxidant, antimicrobial [11], anti-inflammatory [12], antidiabetic [13], and antitumor [14] activities, among other uses.

Taxonomically, some species have been transferred from* Cassia* genus to* Senna* genus [15]. This taxon currently comprises 500–600 species [14, 16], of which many have not been yet characterized with respect to their chemical compositions and biological properties as the arboreal species* Senna velutina* (Vogel) H. S. Irwin & Barneby (Fabaceae, Caesalpinioideae). In this context, the aim of the present study was to determine the chemical composition of an ethanol extract of* S. velutina* leaves and to evaluate its antioxidant and antileukemic activities.

## 2. Materials and Methods

### 2.1. Plant Material and Extract Preparation

*S. velutina* leaves were collected following the identification of the plant and authorization of the SISBIO (Sistema de Autorização e Informação em Biodiversidade, permit number 54470-1) in Dourados, Mato Grosso do Sul (S 22°05′545′′ and W 055°20′746′′), Brazil, oven-dried with the air circulation at a temperature of 45 ± 5°C, and then ground in a Willy-type knife mill. An exsiccated sample was deposited in the Herbarium of the Federal University of Grande Dourados, Mato Grosso do Sul, Brazil, with registration number 4665.

The extract was then prepared by macerating the plant material in an ethanol 95% mixture at room temperature in the dark for 7 days. Then, the extract was filtered, and the residue was further extracted twice using the same process. After 21 days, the filtrate was concentrated in a rotary vacuum evaporator (Gehaka, São Paulo, SP, Brazil) to obtain the ethanol extract of* S. velutina* leaves (ESV). The dry extract yield was 29%, calculated using the following formula: extraction yield (%) = (weight of the freeze-dried extract × 100)/(weight of the original sample). The ESV was stored at −20°C protected from light.

### 2.2. Chemical Analysis

The extract was analyzed by Ultra Fast Liquid Chromatography (UFLC) (Shimadzu) coupled to Diode Array Detector (DAD) (240–800 nm, Shimadzu) and electrospray ionization time-of-flight (ESI-QTOF-micrOTOF QII) (operating in positive and negative mode, 120–1200 Da, Bruker Daltonics). A C-18 column was used (Kinetex, 2.6 *μ*m, 150 × 2.2 mm, Phenomenex) protected by a guard column of the same material. The mobile phase was as follows: water (solvent A) and acetonitrile (solvent B) both with 0.1% of formic acid in a gradient of 0–2 min 3% B, 2–25 min 3–25% B, and 25–40 min 25–80% B followed by washing and reconditioning of the column (8 minutes). The flow rate was 0.3 mL/min and 1 *μ*L (1 mg/mL) of extract was injected. The other micrOTOF-QII parameters were as follows: temperature, 200°C; N_2_ drying gas flow rate, 9 L/min; Nebulizer, 4.0 bar; capillary voltage, −3500 V (negative) and +4500 V (positive); and internal calibration with TFA-NA injected at the end of the chromatographic analysis. The rutin and epicatechin standards were obtained from Sigma-Aldrich with a purity of ≥95%.

### 2.3. Antioxidant Activity

#### 2.3.1. DPPH Free Radical Scavenging Activity

The 2,2-diphenyl-1-picrylhydrazyl (DPPH) radical scavenging activity of ESV was evaluated as described in D. Gupta and R. K. Gupta [17], with modifications. In this assay, 0.2 mL of ESV at different concentrations (1–1000 *μ*g/mL) was added to 1.8 mL of DPPH solution (0.11 mM) in 80% ethanol. The mixture was incubated for 30 minutes at room temperature in the dark. Absorbance at 517 nm was then measured spectrophotometrically. Ascorbic acid and butylhydroxytoluene (BHT) were used as reference antioxidants. As a control, 0.2 mL of solvent used to dilute the extract was added to 1.8 mL of DPPH solution (0.11 mM) in 80% ethanol. Two independent experiments were performed in triplicate. The percentage inhibition was calculated relative to the control using the following equation:

(1) inhibition of DPPH radical%=1−AbssampleAbscontrol×100.

#### 2.3.2. Inhibition of Lipid Peroxidation in Human Erythrocytes

*(1) Preparation of Erythrocyte Suspension.* After approval by the Research Ethics Committee (Comitê de Ética em Pesquisa, CEP) of the University Center of Grande Dourados (UNIGRAN, Brazil (CEP process number: 123/12)), peripheral blood from healthy donors was collected into tubes containing sodium citrate which were then centrifuged at 400 ×g for 10 min. The plasma and leukocyte layer were discarded, and the erythrocytes were washed 3 times with 0.9% sodium chloride solution (NaCl) and centrifuged. Finally, 10% erythrocyte suspension was prepared in 0.9% NaCl solution to attain a 2.5% final concentration for further analysis.

*(2) Hemolytic Activity and Inhibition of Oxidative Hemolysis.* The ability of ESV to protect against lipid peroxidation was evaluated using an antihemolytic assay in human erythrocytes that were incubated with the oxidant 2,2′-azobis-(2-amidinopropane) dihydrochloride (AAPH), a thermoinducible initiator of lipid peroxidation, as described by Campos et al. [18]. To evaluate hemolytic activity and inhibition of oxidative hemolysis, erythrocytes were preincubated with either ESV or ascorbic acid (25–125 *μ*g/mL) at 37°C for 30 min before the addition of 0.5 mL of either 0.9% NaCl or 50 mM AAPH (dissolved in 0.9% NaCl). In the assay, 1% ethanol was used as a solvent control, while 0.9% NaCl was used as the baseline hemolysis control. Total hemolysis was induced by incubating erythrocytes with distilled water. Samples were incubated at 37°C with regular stirring. The extent of hemolysis was determined at 60-minute intervals for an incubation period of 5 h. Tubes were centrifuged at 700 ×g for 5 minutes; 0.2 mL of the supernatant was collected and added to 1.8 mL of 0.9% NaCl for spectrophotometric reading at 540 nm. The hemolysis percentage was calculated using the formula *A*/*B* × 100, where *A* is the sample absorbance and *B* is the absorbance of the total hemolysis sample. Three independent experiments were performed in triplicate. 

*(3) Malondialdehyde (MDA) Measurements.* The inhibition of malondialdehyde (MDA) production, which is a byproduct of lipid peroxidation, was evaluated according to the method described by Campos et al. [18]. Erythrocytes were preincubated at 37°C for 30 min with either ESV or ascorbic acid (25–125 *μ*g/mL) before the addition of 0.5 mL of 50 mM AAPH solution. The mixtures were incubated at 37°C with regular stirring; 1% ethanol was used as a negative control. The MDA concentration was determined at 60-minute intervals for a total of 5 h. To determine the MDA concentration, samples were centrifuged at 700 ×g for 5 min; 0.5 mL of each supernatant was collected and transferred to a tube containing 1 mL of 10 nM thiobarbituric acid (TBA), dissolved in 75 mM monobasic potassium phosphate buffer at pH 2.5. The standard controls used were 500 *μ*L of a 20 mM MDA solution and 1 mL of TBA. Samples were incubated at 96°C for 45 min and allowed to cool before adding 4 mL of n-butyl alcohol and centrifuging at 1600 ×g for 5 min. The resulting supernatant was read at 532 nm in a spectrophotometer. Two independent experiments were performed in triplicate. Sample MDA levels were expressed in nM/mL, according to the following formula:

(2) $$\begin{array}{c} \text{MDA}={Abs}_{sample}\times \left(\;\frac{20\times 220.32}{{Abs}_{standard}}\;\right). \end{array}$$

### 2.4. Cytotoxic Activity

#### 2.4.1. Cell Culture

Leukemia human cell lines Jurkat and K562 were cultivated in RPMI 1640 (Sigma-Aldrich, Germany) culture medium, supplemented with 10% fetal bovine serum, 100 U/mL penicillin (Sigma-Aldrich, Germany), and 100 *μ*g/mL streptomycin (Sigma-Aldrich, Germany). Cells were cultured in a humidified incubator containing 5% CO_2_ at 37°C.

#### 2.4.2. Cytotoxicity and Cell Death Profile

Cytotoxic activity and the cell death profile were evaluated according to the method described by Paredes-Gamero et al. [19]. Leukemic cells were seeded at 10^5^ cells/mL in 96-well microplates and treated with ESV (0–100 *μ*g/mL) for 24 h. Then, the cells were centrifuged 600 ×g for 6 min and resuspended in binding buffer (0.14 M NaCl, 2.5 mM CaCl_2_, 0.01 M HEPES, and pH 7.4) and incubated at room temperature with 1 *μ*L of Annexin V-FITC (BD Biosciences, San Diego, CA, USA) and 5 *μ*g/mL propidium iodide (PI) Becton Dickinson, USA for 20 min. Sample analysis was performed using Accuri C6 flow cytometer (Becton Dickinson, San Diego, CA, USA), with acquisition of 3,000 events.

#### 2.4.3. Measurement of Mitochondrial Membrane Potential

To evaluate the possible effects of ESV on mitochondrial membrane potential, leukemic cells were incubated with the fluorescent marker JC-1 (5,5′,6,6′-tetrachloro-1,1′,3,3′-tetraethylbenzimidazolylcarbocyanine iodide; Molecular Probes, Eugene, OR, USA) according to the method described by Moraes et al. [20]. JC-1 probe accumulates in mitochondria in a potential-dependent manner. Viable cells with high mitochondrial membrane potential are stained red. Upon reduction of the mitochondrial membrane potential, cells appear green. In this assay, cells were seeded into 24-well plates (10^5^ cells/mL) containing supplemented media and were then incubated with 27.6 *μ*g/mL (Jurkat cells) or 67.5 *μ*g/mL (K562 cells) ESV for 24 h. The cells were then centrifuged and incubated with JC-1 (1 *μ*g/mL) for 15 min at room temperature. Fluorescence readings were performed in a FACSCalibur flow cytometer using CellQuest software (Becton Dickinson, San Diego, CA, USA). A total of 10,000 events were collected per sample.

#### 2.4.4. Caspase-3 Activity

Caspase-3 activity was assessed according to the method described by Moraes et al. [20], with minor modifications. Caspase activity was measured by flow cytometer. Leukemic lineages were treated with ESV (27.6 *μ*g/mL) in 24-well microplates (10^5^ cells/mL) for 24 h. Then the cells were fixed with 2% paraformaldehyde in PBS for 30 min and permeabilized with 0.01% saponin for 15 min at room temperature. Next, the cells were incubated for 1 h at 37°C with anti-cleaved-caspase 3-FITC antibody (Becton Dickinson, USA). After incubation for 40 min, the fluorescence was analyzed by Accuri C6 flow cytometer (Becton Dickinson, USA). A total of 10,000 events were acquired.

#### 2.4.5. Intracellular Calcium and Pan-Caspase Inhibitors

The roles of intracellular calcium and caspases in ESV-promoted cytotoxicity were evaluated according to Bechara et al. [21], with minor modifications. Jurkat cells were pretreated for 1 h at 37°C under a 5% CO_2_ atmosphere with either the intracelullar calcium chelator BAPTA-AM or the pan-caspase inhibitor Z-VAD-FMK; ESV (27.6 *μ*g/mL) was then added to the cells and allowed to incubate for 24 h. Control and treated cells were resuspended in culture medium containing 0.05% trypan blue and were counted in a hemocytometer chamber to determine cell viability (trypan blue exclusion assay).

#### 2.4.6. Cell Cycle Phases

Distribution of cell cycle was determined by PI staining and flow cytometry analysis. Leukemic lineages (10^5^ cells/mL) were treated with ESV (27.6 *μ*g/mL) for 24 h and then were fixed and permeabilized as previously described and treated with 4 mg/mL RNase (Sigma-Aldrich, Germany) for 45 min at 37°C. For DNA labeling, cells were incubated with 5 *μ*g/mL of PI (Sigma Aldrich, Germany). Percentages of cells within cell cycle compartments (G1, S, and G2/M) were determined by Accuri C6 flow cytometer (Becton Dickinson, USA). A total of 10,000 events were acquired.

### 2.5. Statistical Analyses

The data are shown as the mean ± standard error of the mean (SEM) and were analyzed for statistical significant differences between the groups using Student's* t*-test for comparison between two groups and one-way analysis of variance (ANOVA) followed by Dunnett's test for comparison of more than two groups using Prism 5 GraphPad Software. The results were considered significant when *P* < 0.05.

## 3. Results

### 3.1. Chemical Composition of ESV

The metabolites in ESV were identified by interpreting the UV absorption and mass spectra and comparing them with data in the literature. When available, compounds were compared with authentic standards for confirmation.

The identification of compound 2, with* m/z* 305.0660 [M-H]^−^, was based on the fragmentation pattern of epigallocatechin proposed by Dou et al. [22], namely,* m/z* 261 [M-CO_2_]^−^,* m/z* 219 [M-C_4_H_6_O_2_]^−^,* m/z* 179 [M-C_6_H_6_O_3_]^−^,* m/z* 167 [M-C_7_H_6_O_3_]^−^, and* m/z* 165 [M-C_7_H_8_O_3_]^−^, and also on the detection of UV absorption at 270 nm.

Compounds 4, 8, and 9 showed UV absorption patterns that were characteristic of flavonols (270 and 340 nm). Their fragmentation patterns were consistent with those of heteroside derivatives of kaempferol, whose main fragment consists of* m/z* 285 [C_15_H_9_O_6_]^−^. Several compounds of this class have been described in* Senna* genus [23, 24].

**Figure 1:**
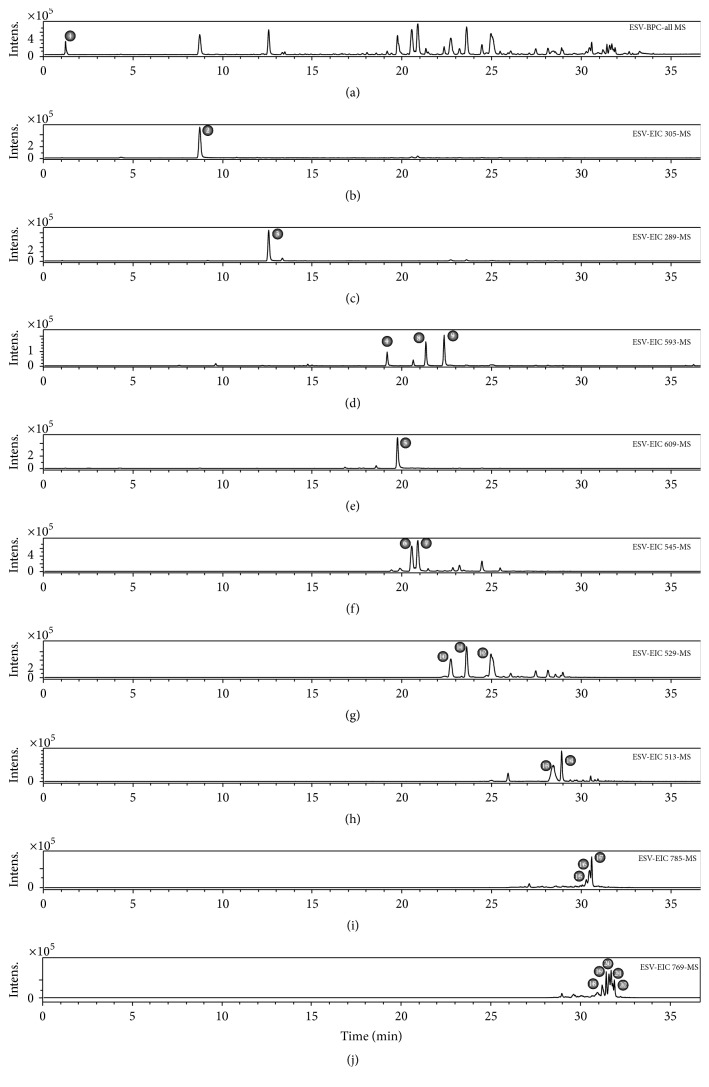
UFLC-DAD-ESI-QTOF-micrOTOF QII chemical profiling (negative mode) of an ethanol extract of* S. velutina* leaves. (a) Base peak chromatograms (BPC). (b) Extract ion chromatogram (EIC) of* m/z* 305. (c) EIC of* m/z* 289. (d) EIC of* m/z* 593. (e) EIC of* m/z* 609. (f) EIC of* m/z* 545. (g) EIC of* m/z* 529. (h) EIC of* m/z* 513. (i) EIC of* m/z* 785. (j) EIC of* m/z* 769.

Dimeric (compounds 6, 7, and 10–14) and trimeric (compounds 15–22) proanthocyanidins, comprising cassiaflavan, afzelechin, epicatechin, epigallocatechin, and naringenin subunits, were also observed. These derivatives, although rare in nature, are often reported in* Senna* genus; some authors consider them to be chemical markers for the genus [25–27]. The UV absorption maxima at 280 nm and the increase in reverse-phase retention time concomitant with decreased hydroxyl group content or increased degree of polymerization are in agreement with the study by Callemien and Collin [28]. Fragmentation patterns obtained by retro-Diels-Alder (RDA) fission, heterocyclic ring fission (HRF), and quinone methide (QM) can be used to characterize the subunits that make up proanthocyanidins [29]. A complete discussion on the elucidation of this class of compounds may be found in the literature [29, 30]. Thus, based on retention times, fragmentation profiles, and comparisons with previously published data, 22 compounds in ESV were characterized (Figure 1 and Table 1).

### 3.2. DPPH Free Radical Scavenging Activity

ESV was able to scavenge the DPPH free radical, with a 2.5-fold higher IC_50_ and maximum activity values relative to ascorbic acid; however, these values were lower than those obtained with BHT (Table 2).

### 3.3. Hemolytic Activity and Inhibition of Oxidative Hemolysis

**Figure 2:**
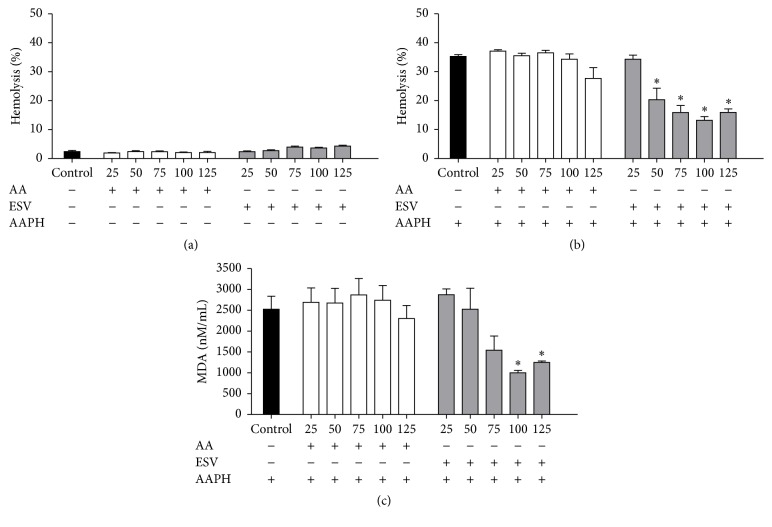
Hemolysis and MDA content in human erythrocytes incubated for 5 hours with ascorbic acid (AA) and ESV (50–125 *μ*g/mL). (a) Hemolytic activity of ESV in the absence of AAPH. (b) Antihemolytic activity after addition of AAPH. (c) Malondialdehyde (MDA) concentration (nM/mL) after addition of the oxidizing agent. ^*∗*^ *P* < 0.05 compared to the AAPH-only control (erythrocytes incubated with oxidant only).

Over the range of tested concentrations, ESV showed no hemolytic activity in human erythrocytes, as no hemolysis was observed after up to 5 h of incubation (Figure 2(a)).

The control antioxidant, ascorbic acid, was able to protect erythrocytes from hemolysis for up to 4 h of incubation when they were exposed to the oxidant AAPH (data not shown). ESV was able to protect erythrocytes for 5 h over the tested 50–125 *μ*g/mL concentration range (Figure 2(b)), demonstrating its powerful antihemolytic activity.

### 3.4. MDA Measurements

The degree of protection conferred by ESV against AAPH-induced lipid peroxidation in human erythrocytes was evaluated by measuring MDA levels. At ESV concentrations of 100 and 125 *μ*g/mL, the MDA levels were decreased throughout the course of the assay (data not shown) and after 5 h of incubation (Figure 2(c)).

### 3.5. Cytotoxic Activity and Cell Death Profile

**Figure 3:**
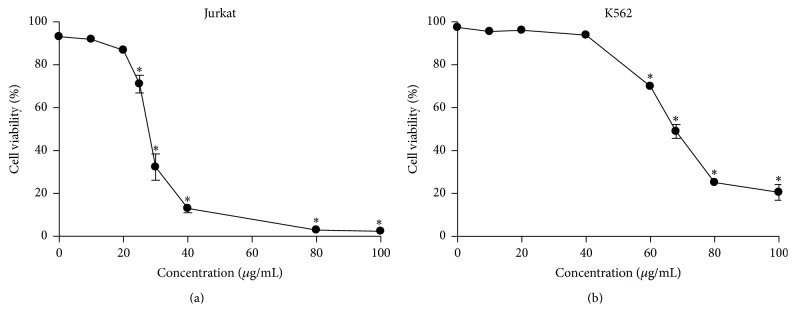
Viability of leukemic Jurkat (a) and K562 (b) cells after treatment with different concentrations of ESV. ^*∗*^ *P* < 0.05 compared to the untreated control group.

**Figure 4:**
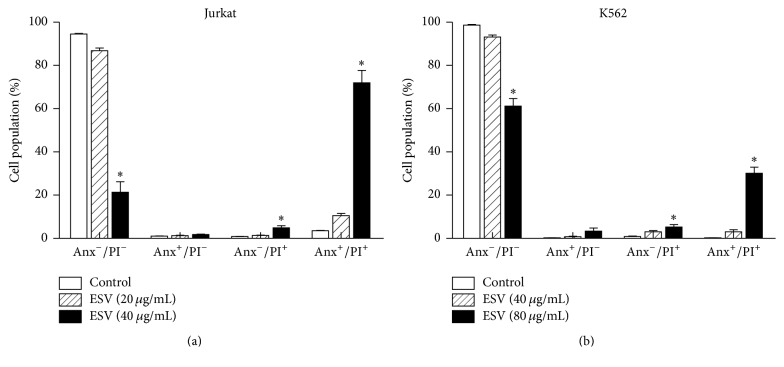
Cell death profiles of ESV-treated Jurkat (a) and K562 (b) cells. Anx^−^/PI^−^, viable cells; Anx^+^/PI^−^, apoptotic cells; Anx^−^/PI^+^, necrotic cells; and Anx^+^/PI^+^, late apoptotic cells. ^*∗*^ *P* < 0.05 compared to the respective control groups.

ESV promoted cell death in both tested cell lines. IC_50_ values indicated that ESV was more effective in Jurkat cells than in the erythroleukemic cell line K562 (IC_50_ = 27.6 *μ*g/mL and 67.5 *μ*g/mL, resp.) (Figures 3(a) and 3(b)). ESV treatment promoted double staining in both cell lines. This type of death was evident in 71.9 ± 5.7% of Jurkat cells and 30.1 ± 2.8% of K562 cells after treatment with 40 and 80 *μ*g/mL of extract, respectively (Figures 4(a) and 4(b)).

### 3.6. Mitochondrial Membrane Potential

**Figure 5:**
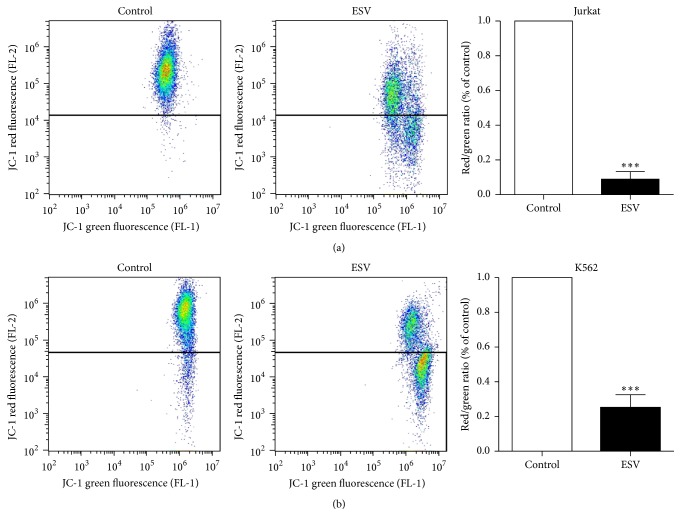
Mitochondrial membrane potential of leukemic Jurkat (a) and K562 (b) cells treated with different ESV concentrations. ^*∗∗∗*^ *P* < 0.0001 compared to the untreated control group.

The mitochondrial membrane potentials of Jurkat and K562 leukemic cells decreased after 24 h of incubation with ESV, as evidenced by a decrease in red fluorescence and an increase in green fluorescence compared to untreated cells. The mitochondrial membrane potential was reduced by 91.0 ± 4.3% in Jurkat cells and by 74.7 ± 7.3% in K562 cells after treatment with 27.6 and 67.5 *μ*g/mL of extract, respectively (Figures 5(a) and 5(b)).

### 3.7. Caspase-3 Activity

**Figure 6:**
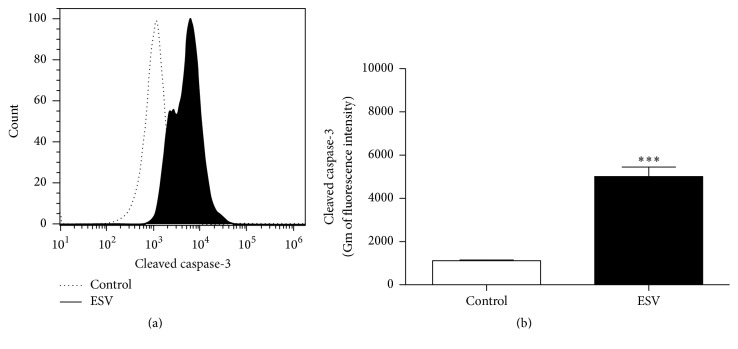
Histogram (a) and representative graph (b) of caspase-3 activation in ESV-treated Jurkat cells. ^*∗∗∗*^ *P* < 0.0001 compared to the untreated control group.

The Jurkat cell line, which was more sensitive to ESV activity, was used to investigate ESV-promoted cell death mechanisms. The fluorescence histogram (Figure 6(a)) showed a rightward shift (greater fluorescence values), indicating the activation of the apoptosis-inducing enzyme caspase-3. Cleaved caspase-3 levels increased 4.5-fold in ESV-treated cells relative to untreated cells (Figure 6(b)).

### 3.8. Pan-Caspase Inhibition and Intracellular Calcium Chelation

**Figure 7:**
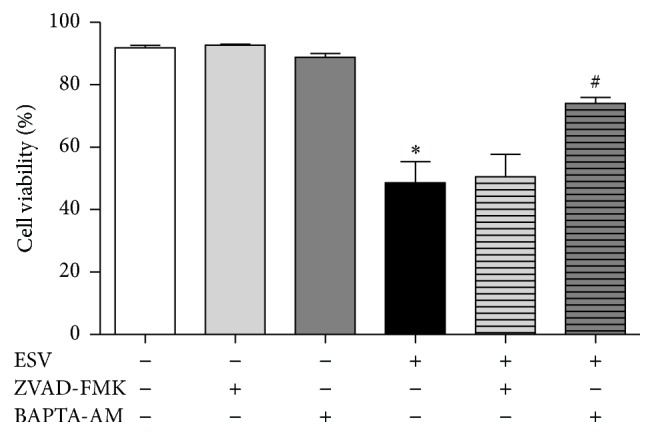
Involvement of caspases (via the pan-caspase inhibitor Z-VAD-FMK) and intracellular calcium (using the chelator BAPTA-AM) in ESV-induced Jurkat cell cytotoxicity. ^*∗*^ *P* < 0.05 compared to the untreated control group. ^#^ *P* < 0.05 compared to the ESV group.

Jurkat cells were preincubated with the pan-caspase inhibitor Z-VAD-FMK to assess whether ESV cytotoxicity was mainly mediated by caspase activation. Z-VAD-FMK pretreatment did not decrease ESV-induced cell death (Figure 7). However, the intracellular Ca^2+^ chelator BAPTA-AM partially inhibited ESV-induced death in Jurkat cells (Figure 7).

### 3.9. Cell Cycle Phases

**Figure 8:**
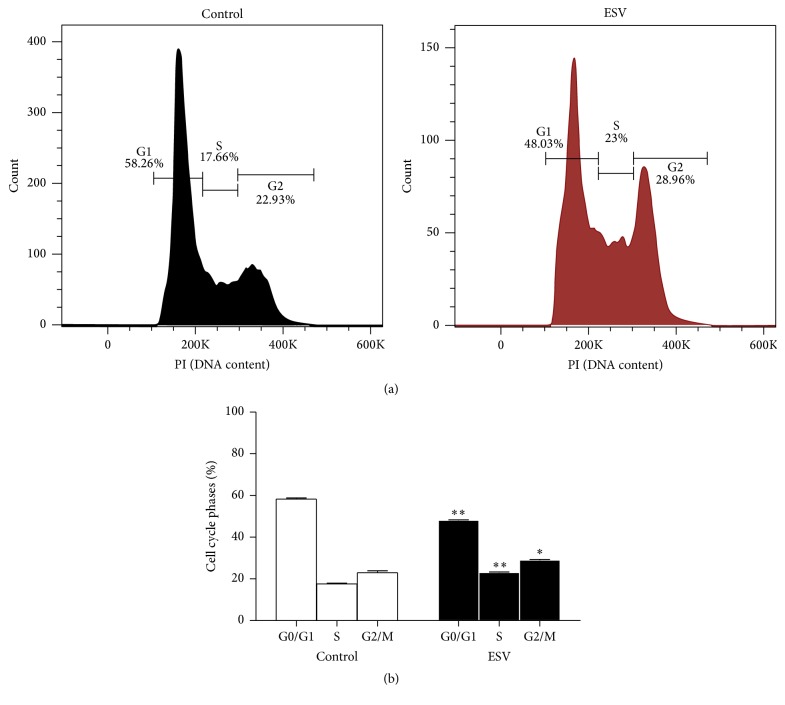
Histogram (a) and representative graph (b) of cell cycle distribution after 24 h of treatment with ESV. ^*∗*^ *P* < 0.05 and ^*∗∗*^ *P* < 0.001 compared to the untreated control group.

Histograms were used to show the distributions of cell cycle phases in control and ESV-treated Jurkat cells after 24 h of incubation (Figure 8(a)). The results show a decreased portion of cells in G0/G1 phase (17.4 ± 0.6%) and increased portions of cells in S and G2/M phases (30.7 ± 1.8% and 26.5 ± 1.9%, resp.) (Figure 8(b)). These results indicate that ESV inhibits the progression of cell cycle transitions.

## 4. Discussion

Brazilian biodiversity is rich in active compounds with high potential for development of new therapeutic drugs, particularly antioxidants and anticancer agents. Several plant species found in Brazil have been characterized for their antioxidant and cytotoxic activities in several tumor cell lines [31–33].

In the present study, an ethanol extract of* S. velutina* leaves exhibited antioxidant activity and showed cytotoxic effects against two leukemic cell lines, Jurkat and K562. The antioxidant activity of ESV was demonstrated in AAPH-incubated human erythrocytes; DPPH free radical scavenging and inhibition of lipid peroxidation led to decreases in oxidative hemolysis and malondialdehyde production. This activity of ESV is likely related to the presence of flavone derivatives with antioxidant activity, such as epigallocatechin, epicatechin, rutin, kaempferol glycosides, and dimeric and trimeric proanthocyanidins, in the leaves [34–37].

Flavonoids can donate hydrogen atoms to radicals, protecting against lipid peroxidation, and this ability is associated with the presence of a dihydroxylated B-ring [38]. Similar to other phenolic compounds, the antioxidant activity of flavonoids is ascribed to the presence of free hydroxyl groups in the molecule, and the level of antioxidant activity increases concomitantly with the number of hydroxyl groups [39].

Excess free radicals in the body can promote not only lipid peroxidation but also oxidative DNA damage, leading to the development of early stages of mutagenesis and carcinogenesis [40, 41]. Thus, compounds with antioxidant properties have fundamental roles in preventing diseases such as cancer.

The cytotoxic activity of ESV against leukemic lines was evaluated with an Annexin/PI-stain cell death assay. Furthermore, activation of caspase-3 was observed but was not confirmed to be a main mechanism of cell death when the pan-caspase inhibitor Z-VAD-FMK was used. Different extracts can promote several different death mechanisms simultaneously because of their different compositions. This fact complicates the analysis and identification of specific cell death pathways.

The ESV-induced decrease in mitochondrial membrane potential is consistent with the observed cytotoxic effect of ESV against leukemic cells. The changes in potential are likely due to increased mitochondrial membrane permeability arising from an increase in intracellular calcium levels, a characteristic of necrotic cell death [42, 43].

The present study demonstrated the involvement of calcium in cell death, as the reduced cell viability of extract-treated cells was reversed by incubation with the calcium chelator BAPTA-AM. High calcium levels promote the opening of mitochondrial permeability transition pores; these pores are nonselective and thus release the contents of the intermembrane space of the mitochondrion [44].

Another mechanism of the ESV-induced cytotoxicity against Jurkat cells consists of cell cycle arrest. ESV promoted a decrease in the number of cells in G0/G1 phase and an increase in the number of cells in S and G2 phases. Established anticancer drugs, such as cisplatin and doxorubicin, exert a similar profile change in the tumor cell cycle [14, 45]. Mueller et al. [46] observed that cisplatin is likely to be active in G2/M phases because cells in these phases are more sensitive to DNA damage, as DNA repair mechanisms are less active than in G1/S phases. Flavonoids are phytochemicals that are known to induce cell cycle arrest by decreasing cellular levels of cyclin B and cyclin-dependent kinase 1, which are responsible for controlling cell cycle progression between S and M stages [47]. Furthermore, one of the major anticancer mechanisms ascribed to flavonoids is their ability to induce cell cycle changes in tumor cell lines [48]. Thus, cyclin-dependent kinase inhibitors (CDKIs) have generated great interest for their ability to arrest the tumor cell cycle and prevent tumor cell proliferation; such cell cycle arrest may be the main mechanism through which ESV operates [49]. However, plant extracts are natural products with complex chemical composition, and biologically active compounds in extracts may act alone or synergistically through different pathways.

In conclusion, an ethanol extract of* S. velutina* leaves exhibited antioxidant activity and showed cytotoxic effects on leukemic cells by activating intracellular calcium and caspase-3, decreasing mitochondrial membrane potential, and arresting the cell cycle in S and G2 phases.

## Figures and Tables

**Table 1:** Compounds identified in ESV by UFLC-DAD-ESI-QTOF-micrOTOF QII.

ID	Time (min)	UV	[M-H]^−^ (*m*/*z*)	Molecular formula	Error (ppm)	MS/MS	Compound
1	1.1	—	341.1086	C_12_H_20_O_11_	0.6	341: 179	Sugar derivative
2	8.6	270	305.0660	C_15_H_14_O_7_	2.3	305: 261, 219, 179, 167, 165	Epigallocatechin
3	12.5	280	289.0714	C_15_H_14_O_6_	1.3	289: 245, 205, 203	Epicatechin
4	19.1	270/346	593.1524	C_27_H_30_O_15_	2.1	593: 447, 285	Kaempferol-*O*-hexoside-deoxyhexoside
5	19.7	270/346	609.1450	C_27_H_30_O_16_	1.9	609: 463, 301	Rutin
6	20.5	280	545.1440	C_30_H_26_O_10_	2.5	545: 305, 239, 219, 167, 165	Cassiaflavan-epigallocatechin
7	20.8	280	545.1440	C_30_H_26_O_10_	2.5	545: 305, 239, 219, 167, 165	Cassiaflavan-epigallocatechin
8	21.2	268/338	593.1522	C_27_H_30_O_15_	1.6	593: 447, 285	Kaempferol-*O*-hexoside-deoxyhexoside
9	22.3	270/342	593.1521	C_27_H_30_O_15_	1.5	593: 447, 285	Kaempferol-*O*-hexoside-deoxyhexoside
10	22.6	280	529.1489	C_30_H_26_O_9_	2.9	529: 289, 245, 239, 203	Cassiaflavan-epicatechin
11	23.5	280	529.1484	C_30_H_26_O_9_	3.7	529: 289, 245, 239, 203	Cassiaflavan-epicatechin
12	24.9	280	529.1489	C_30_H_26_O_9_	2.9	529: 267, 257, 239, 151	Naringenin-afzelechin
13	28.3	280	513.1551	C_30_H_26_O_8_	0.8	513: 267, 255, 239	Cassiaflavan-afzelechin
14	28.8	280	513.1541	C_30_H_26_O_8_	2.8	513: 267, 255, 239	Cassiaflavan-afzelechin
15	30.2	280	785.2266	C_45_H_38_O_13_	3.4	785: 435, 305, 239	Cassiaflavan-cassiaflavan-epigallocatechin
16	30.4	280	785.2285	C_45_H_38_O_13_	1.7	785: 435, 305, 239	Cassiaflavan-cassiaflavan-epigallocatechin
17	30.5	280	785.2255	C_45_H_38_O_13_	2.0	785: 435, 305, 239	Cassiaflavan-cassiaflavan-epigallocatechin
18	31.1	280	769.2310	C_45_H_38_O_12_	2.6	769: 529, 419, 289	Cassiaflavan-cassiaflavan-epicatechin
19	31.3	280	769.2303	C_45_H_38_O_12_	1.6	769: 529, 419, 289	Cassiaflavan-cassiaflavan-epicatechin
20	31.5	280	769.2295	C_45_H_38_O_12_	0.6	769: 377, 267, 239	Cassiaflavan-naringenin-afzelechin
21	31.7	280	769.2310	C_45_H_38_O_12_	2.6	769: 377, 267, 239	Cassiaflavan-naringenin-afzelechin
22	31.8	280	769.2297	C_45_H_38_O_12_	0.9	769: 377, 267, 239	Cassiaflavan-naringenin-afzelechin

**Table 2:** IC_50_ and maximal DPPH radical scavenging activity of standard antioxidants and of ESV.

Sample	IC_50_ (*µ*g/mL)	Maximal inhibition	
		%	*µ*g/mL
Ascorbic acid	2.6 ± 0.8	90.9 ± 1.6	10
BHT	21.3 ± 1.2	92.4 ± 1.2	250
ESV	6.3 ± 1.3	92.4 ± 0.4	25

Values are means ± SEM.

## Abbreviations

AA: Ascorbic acid
AAPH: 2,2′-Azobis-(2-amidinopropane) dihydrochloride
Abs: Absorbance
Anx: Annexin V-FITC
BHT: Butylhydroxytoluene
Ca^2+^: Calcium
CaCl_2_: Calcium chloride
DAD: Diode Array Detector
DPPH: 2,2-Diphenyl-1-picrylhydrazyl
ESI-QTOF-micrOTOF QII: Electrospray ionization time-of-flight
ESV: Ethanol extract of* S. velutina* leaves
JC-1: 5,5′,6,6′-Tetrachloro-1,1′,3,3′-tetraethylbenzimidazolylcarbocyanine iodide
MDA: Malondialdehyde
NaCl: Sodium chloride
PI: Propidium iodide
SEM: Standard error of the mean
TBA: Thiobarbituric acid
UFLC: Ultra Fast Liquid Chromatography.
